# Economics and a Vocation

**Published:** 1918-11-02

**Authors:** 


### THE NURSES' DAY AND FUTURE OUTLOOK.

### Economics and a Vocation.

Many people insist that no woman should become a nurse unless she has as a preliminary qualification what is known as a vocation. The word is really borrowed from religion. When men or women contemplate vows of poverty and celibacy it is extremely important that so serious a step should not be taken by way of whim or under the spell of excitement. Hence the extraordinary precautions taken to discover that there is in verity a call, a burning zeal for service and sacrifice eclipsing the glamour of youth and pleasure. I do not know that any other field of labour can bear comparison with this. Mariners respond to the call of the sea; the Italian vendetta is commonly regarded as the call of the blood; woman responds to the call of motherhood. And although clerks often like the desk, farmers the plough, and literary men the pen, the word "vocation" is seldom used in connection with their work, and properly so. What usually happens is that early up-bringing, environment, and natural talent result in congenial occupation, and as the worker succeeds he gets to like it better, even if it consists to a large extent of drudgery.

The question is therefore put, Does it necessarily follow that a thoroughly efficient nurse who loves her work is inspired by Nature, and, in the highest sense, possessed of a vocation? I shall not attempt to answer this far-reaching question myself, for it has already been answered by a higher authority, one whose dictum is not likely to be challenged. I refer to Florence Ninghtingale, who with a touch of genius summed up the situation in a single sentence: "*Every woman is a nurse*!" Here is wrapped up a whole volume of science, philosophy, and truth. Latent or developed, the call is there, an essential part of a woman's being, and at some period of her life she responds to it with all the fervour of her soul. No particular woman or school of women was contemplated by the poet when he sang:

"When pain and anguish rend the brow
A ministering angel thou!"

Florence Nightingale was not a dreamer, but one of the most practical of women. None has ever been more emphatic on the importance of first-class training. It is sixty years since she wrote her "Notes on Nursing," and it is no exaggeration to say that she was a century ahead of her time. I am convinced that in no modern institution, however well equipped and up to date, does so exacting a standard of training prevail as that which she created in those early days, when skilled nursing was scarcely known. To this interesting subject I hope in the future to refer. What I desire at the moment to emphasise is that the Mother of the Trained Nurse, with that perception which is born of genius, settled for ever and by first principles the vocation question. Her answer carries conviction, and clears the air of mist and difficulty. Every woman is a nurse. There is no more to be said, except perhaps this, that the army of women who are not nurses, and who have during the war responded to the call of the Red Cross, is a standing endorsement of the statement.

Now, it does not follow that everybody who has a vocation cultivates it as a means of livelihood. Scores of highly talented musicians spend their days as clerks, teachers, merchants, statesmen. Mr. Balfour can make the midnight air ring with music. But he can do other things, for, as he confessed on one occasion, the remorse of his life was that he had not begun to play golf sufficiently early to acquire the skill that he would like to possess! The question, therefore, arises, Why does not everybody devote his life to the call that Nature makes? The sordid answer is that it does not pay. In religion, money does not count. On the contrary, poverty is a condition of conventual service. Religion apart, however, all other classes of the community are affected by the economic question in selecting a career. Hence, for example, the universal demand for better employment conditions for teachers. Literature, the Bar, the Civil Service, and even the Church offer bigger prizes, and, therefore, ideal teachers, men and women with a vocation, make the profession a stepping-stone to higher things. Rudyard Kipling began life as a schoolmaster. The nursing profession is paid according to a beggarly standard, and the best women are accordingly choosing other and more remunerative fields of service, leaving the residue to nurse the nation's sick. There is a shortage of nurses. There has always been a shortage of nurses, and for the same reason. Women never did and never will abandon the joy of life to serve in a condition of perpetual bondage at a bare subsistence wage no matter how loud the call. A thirteen hours' day, no one day's rest in seven, no weekly half-day off duty, no pension—these are hard and strong counterbalancing facts of existence even to a call that is instinctive. And if you want authority come back to the fountain. The economics of nursing were not overlooked or forgotten by Florence Nightingale. Bitterly complaining of the shortage of nurses sixty years ago, she says: "I have had during the past three years several hundreds of applications to recommend qualified matrons or superintendents of institutions—qualified missionaries or parish nurses—qualified sick nurses for private families, for hospitals, for workhouses." Unable to supply the demand, and, seeking for the true reason for the shortage, she points out that "only about one-third of these offered ample remuneration."

Have we grown? I leave this question to be answered by the reader. Personally, I see scant progress, and, moreover, little prospect of advancement so long as "tradition shrouds our minds," as A. H. I. observes in her very able letter of last week. This letter is like a breeze from the prairie—a tonic to weary women, and if any still harbour lingering doubts as to the forsaking of high ideals, or disloyalty to a great profession, let them find assurance and consolation in the words of Florence Nightingale. I ask for no higher standard. IERNE.

